# Multi-organ Thromboembolic Crisis: A Case Report of Concomitant Stroke, Myocardial Infarction, and Pulmonary Embolism

**DOI:** 10.7759/cureus.63288

**Published:** 2024-06-27

**Authors:** Satheesh Gunaga, Joanna V Fourtounis, Kirby W Swan, Seth P Butler, Abe Al Hage

**Affiliations:** 1 Emergency Medicine, Henry Ford Wyandotte Hospital/Envision Healthcare/Michigan State University College of Osteopathic Medicine, Wyandotte, USA; 2 Emergency Medicine, Henry Ford Wyandotte Hospital, Wyandotte, USA; 3 Emergency Medicine, Michigan State University College of Osteopathic Medicine, East Lansing, USA; 4 Emergency Medicine, University at Buffalo, Buffalo, USA

**Keywords:** thromboembolic disease, primary palliative care, endovascular procedures, emergency medicine, st-segment elevation myocardial infarction (stemi), multi-organ thromboembolic crisis, thrombotic complications, pulmonary embolism, stroke, myocardial infarction

## Abstract

Management of acute coronary syndrome (ACS), cerebrovascular accident (CVA), and pulmonary embolism (PE) necessitates prompt intervention, as delayed treatment may lead to severe consequences. Each of these conditions presents significant challenges and carries a high risk of morbidity and mortality. We present the case of an 86-year-old female with a history of stage 4 urothelial carcinoma metastasized to the lungs, who presented to the emergency department (ED) with acute ischemic stroke (AIS), ST-segment elevation myocardial infarction (STEMI), and bilateral PE. We propose the term “multi-organ thromboembolic crisis” (MOTEC) to streamline the communication and management approach for patients experiencing critical thromboembolic events affecting multiple organ systems.

## Introduction

The pathophysiology and management of thromboembolic phenomenon, chronicled in seminal works such as those by Virchow, have been extensively documented throughout modern medical literature [1-3]. According to the 2021 annual report from the Centers for Disease Control and Prevention (CDC), acute coronary syndrome (ACS) and cerebrovascular accidents (CVA) are identified as the first and fifth leading causes of death in the United States, collectively claiming over 850,000 lives annually [4]. Similarly, deep venous thrombosis (DVT) and pulmonary embolism (PE) contribute to 60,000 to 100,000 deaths per year, with approximately one-third of survivors experiencing recurrence within a decade [5].

The identification and management of these patients begins as early as the prehospital setting and continues in the emergency department (ED), with more definitive treatments administered in interventional endovascular labs and intensive care units. Modern emergency medicine has developed sophisticated protocols to address these conditions effectively [6-8]. With advancements in our understanding of these pathological processes, the precision of available interventional treatments has also improved [6-8]. Nonetheless, each of these diagnoses can have severe implications for patients, proving challenging to manage even under optimal circumstances. These challenges are compounded significantly in rare cases where these diseases coexist. We present a case of an 86-year-old female who experienced acute ischemic stroke (AIS), ST-segment elevation myocardial infarction (STEMI), and bilateral PE simultaneously. This constellation of critical thromboembolic events affecting multiple organ systems simultaneously exemplifies what we term “multi-organ thromboembolic crisis” (MOTEC). MOTEC is defined as a rare and complex clinical syndrome wherein critical thromboembolic occlusions occur concurrently across multiple separate organ systems. MOTEC presents unique diagnostic and therapeutic dilemmas due to its multifaceted impact on patient physiology and clinical outcomes. By formally introducing the term MOTEC into the literature, we aim to facilitate clearer communication among healthcare providers and refine management algorithms tailored to address the intricacies of simultaneous multi-organ thromboembolic crises.

## Case presentation

**Figure 1:**
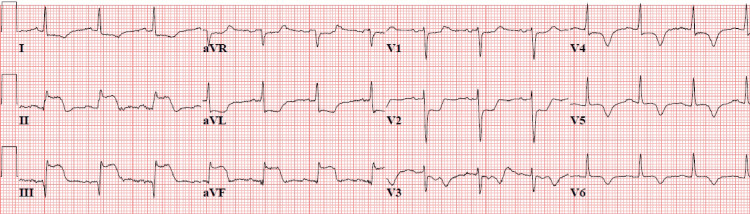
12-lead electrocardiogram showing severe ST-segment elevation myocardial infarction (STEMI) in leads II, III, and aVF with significant reciprocal changes in leads V1, V2, and V3 suggesting inferior STEMI.

An 86-year-old female with a history of stage four urothelial carcinoma with metastasis to the lungs presented to the ED via emergency medical services (EMS) from a nursing home with stroke-like symptoms. Her symptoms included acute onset of confusion, left-sided paralysis with slurred speech, and somnolence with last known well 2.5 hours prior to ED arrival. After speaking with the nursing home staff, it was identified that the patient’s baseline status was alert and oriented x3, able to ambulate with a walker, and did not require much daily assistance. At the time of arrival to the ED, she had a heart rate of 108 beats per minute, blood pressure of 181/85 millimeters of mercury (mmHg), a respiratory rate of 19 breaths per minute, and an oxygen saturation of 96% on room air. Physical examination revealed the presence of total left-sided upper and lower extremity paresis, sensory loss, speech changes, and a right gaze deviation confirming a moderate to severe stroke severity with a National Institutes of Health Stroke Scale (NIHSS) score of 16. Cardiopulmonary examination revealed decreased breath sounds and rales throughout bilateral lung fields with regular tachycardia. At that time, the working diagnosis was focused on severe AIS. Per ED protocol, the patient had a STAT non-contrast computed tomography (CT) head conducted without evidence of hemorrhage, indicating the patient to be a candidate for tissue plasminogen activator (tPA). However, before administration of tPA, further ED workup quickly revealed STEMI on a 12-lead electrocardiogram (ECG) (Figure 1).

**Figure 2:**
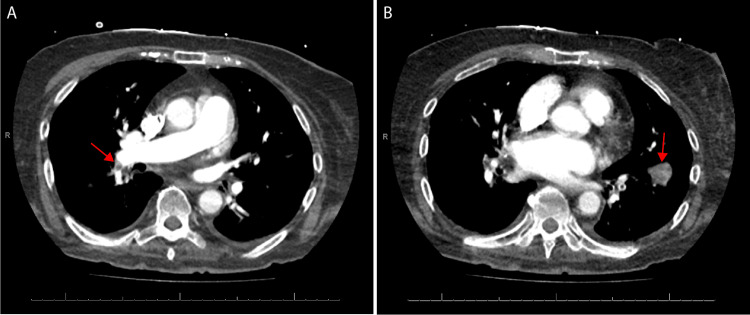
Computed tomography (CT) pulmonary angiogram depicting (A) emboli in the right main pulmonary artery, extending into the right lower lobe and (B) large emboli extending into the left lower lobe.

At this point, in the presence of severe AIS and STEMI, the patient’s differential was reconsidered and prompted the team to order a CT angiogram (CTA) of head, neck, chest, abdomen, and pelvis for concern for possible aortic dissection. It demonstrated potential right carotid artery occlusion and bilateral pulmonary emboli with involvement of the right mainstem pulmonary artery (Figures 2A, 2B).

Given the patient’s terminal stage 4 urothelial carcinoma diagnosis as well as the severity of her multiorgan failure, the case was discussed with both neurology and interventional cardiology teams. The patient was deemed not to be a candidate for tPA, cardiac catheterization or other anticoagulants because of risks associated with hemorrhagic transformation of the ischemic stroke. Her poor prognosis was discussed with family and the consensus was to focus on comfort care measures. The patient was placed into inpatient hospice care, where she died 12 hours later.

## Discussion

Pathological thrombosis arises from an imbalance in the body’s natural coagulation cascade. Previous studies have highlighted genetic deficiencies, mutations, disseminated intravascular coagulation (DIC), and malignancy as primary predisposing factors for thrombosis [9-11]. Cancer, known for its inflammatory and prothrombotic nature, sets off a cascade of events involving tissue factors, inflammatory cytokines, and other cancer pro-coagulant factors. These factors lead to endothelial dysfunction, direct activation of factor X and the extrinsic coagulation cascade, and inhibition of the fibrinolytic system [10]. Other pathological conditions, such as DIC, can also manifest similarly. Triggered by infections, trauma, or multi-organ failure, DIC involves severe systemic activation and depletion of coagulation factors resulting in widespread microvascular thrombosis and bleeding [11]. In contrast, for embolism to occur, there usually is an inciting incident that leads to either displacement and migration of a clot, or an embryonic anatomical variant such as foramen ovale, that favors migration to distant, vulnerable structures. Venous thromboembolism (VTE) ranks as the second most prevalent cause of death in cancer [10]. Our patient had a medical history of advanced metastatic cancer, likely contributing to her severe pro-thrombotic state.

Thrombotic and embolic phenomena are common in emergency medicine. There are many documented MOTEC cases describing concomitant AIS and MI, AIS, and PE, or MI and PE, but despite extensive literature review, the authors were only able to identify two other known cases documenting patients with all three of these thrombotic events occurring simultaneously [12,13]. These MOTEC patients differed notably from our patients. They included a 44-year-old carrier of thrombophilia gene polymorphism and a 68-year-old with a patent foramen ovale. Both of whom survived to hospital discharge [12,13]. We not only hope to bring an additional and distinct case to the literature but also formally propose the novel term MOTEC to best describe such patient presentations in the future. The term MOTEC is defined by the authors as a rare and complex clinical syndrome wherein critical thromboembolic occlusions occur concurrently across multiple separate organ systems. Utilizing a succinct, distinctive term like MOTEC clearly identifies the severity as well as the multi-organ pathologic processes involved in these cases. This term helps promote more effective communication between emergency physicians and multiple clinical subspecialists, to define patient-centered care plans for these complex presentations.

Acute management of AIS and concomitant STEMI or PE are time-sensitive processes whereby delay in treating one may result in detrimental repercussions from another [14,15]. The continued evolution of endovascular and surgical interventions has resulted in significantly improved outcomes for these pathologies [16-18]. Emergent percutaneous coronary interventions (PCI) like balloon angioplasty and drug-eluting stents are the standard of care for STEMI and have dramatically improved mortality and morbidity outcomes [16]. Similar outcome benefits are seen with large vessel occlusion (LVO) ischemic strokes as well as sub-massive/massive PE in properly selected patients undergoing mechanical thrombectomy with or without systemic thrombolytic therapy [17,18]. As endovascular technologies and training continue to advance, the research, clinical practice guidelines, and outcome benefits from these types of interventions will also improve. However, currently, these interventions carry significant expense, carry their own side effect profiles, and require sub-specialty physician access that is often found only at tertiary medical centers or large community-based hospitals.

In the absence of immediate endovascular treatment options, systemic lytic therapy (SLT) remains integral in managing these conditions when found in combination [14,15]. Thrombolytic agents are considered first-line therapy in the acute management of AIS patients presenting within the four-and-a-half-hour thrombolytic window [6]. In cases of massive PE accompanied by hemodynamic instability, SLT has demonstrated reduced mortality rates compared to anticoagulation alone [7]. While the role of SLT in STEMI has diminished significantly over the past three decades with the advent of PCI, it still holds significance in patients identified in critical access hospitals where the anticipated delay to PCI exceeds 120 minutes [8].

In the absence of a standardized treatment algorithm tailored to this MOTEC case, the authors propose the following care pathway [6-8]. Clinical care teams are advised to prioritize the fundamental principles of emergency stroke and STEMI care for patients encountering this clinical scenario. Ideal care begins with early activation of 9-1-1 by the nursing home upon recognizing a change in the patient's mental status, followed by prompt EMS response and field stroke activation, ensuring the ED's “stroke team” is ready upon their arrival. Upon ED arrival, the ED team should focus on verifying that the patient's airway, breathing, and circulation (ABCs) are all intact, along with obtaining a point-of-care glucose level and conducting a quick focused neurological assessment including an NIHSS. If AIS continues to be on the differential and the patient is hemodynamically stable, they should proceed on the EMS stretcher directly to CT with members of the stroke team for an emergent non-contrast CT head and CTA head and neck. As soon as the patient is placed on the CT table, the EMS team can be released and can update the ED stroke team on their field history and findings. The ED stroke team also has a short amount of time to review the patient's electronic medical record, past medical history, and active medications, perform a preliminary read of the non-contrast CT head to rule out large bleeds, and begin to identify thrombolytic inclusion and exclusion criteria. Upon the patient's return to the ED, assessment of the ABCs should be repeated, along with a formal NIHSS and a more complete neurological exam. In patients presenting with AIS, other ancillary blood work and tests such as a 12-lead ECG should be performed but should not delay the administration of thrombolytics. In this case, a 12-lead ECG was performed early and immediately demonstrated a concomitant anterior STEMI and the presence of MOTEC.

At this stage, careful consideration should be given prior to administering thrombolytics, particularly to verify whether the patient may be experiencing aortic root dissection affecting the proximal coronary ostia as well as the more distal cerebral circulation. If there is a legitimate concern for this pathology, the patient should undergo a STAT CTA of the chest and abdomen with a dissection protocol to rule it out. Aortic dissection can extend into the carotid vessels and present with AIS-like symptoms in 6% of cases, with devastating consequences if thrombolytics are administered prior to recognition of this pathology [19]. In this case, the CTA of the chest and abdomen revealed no signs of dissection but did show incidental findings of bilateral PE, further illustrating the extent of MOTEC in this patient.

Early consultation with on-call interventional cardiologists and neurologists is crucial. Medical and interventional care plans should be discussed promptly among subspecialists. After engaging in a deliberate shared decision-making process involving the patient, family, and consultants, it is advisable to contemplate the administration of an initial dose of tPA for AIS at 0.9 mg/kg IV, ensuring the total dose does not surpass 90 mg [6,20]. The recommended protocol involves administering 10% of the total dose as an initial IV bolus over 1 minute, followed by infusion of the remaining dose over 60 minutes [6,14]. Under most circumstances, this AIS tPA dose will prioritize treatment of acute cerebral infarction and the developing penumbra but still leaves other cardiac, neurological, or pulmonary interventional therapies available afterward if indicated. This dose of tPA is also considered safe in the context of managing massive or submassive PE, presenting a secondary benefit of this treatment approach. However, this dose does fall approximately 30% below the required therapeutic index for STEMI tPA dosing but is unlikely to cause harm [8,16]. Even though the American Heart Association/Emergency Cardiovascular Care (AHA/ECC) guidelines identify that acute MI (AMI) within three weeks of AIS is a relative contraindication for tPA in stroke, these same guidelines underscore the absence of significant data to deter its use [6]. These recommendations clearly state that in patients with suspected AIS and STEMI, clinicians can consider tPA dosing for AIS, followed by emergent PCI when accessible [6]. For MOTEC patients suffering simultaneous STEMI, PE, and AIS who are neither candidates for endovascular nor SLT approaches, medical management should focus on some regimen of anticoagulation therapy, such as heparin, warfarin, clopidogrel, aspirin, and risk factor management [8].

Early on and at any point during this treatment pathway, the ED team should pause to verify patient and family goals of care. Upon recognizing the extremely poor prognosis for this patient’s MOTEC and gaining a better understanding of her end-of-life wishes, palliative care consultation was promptly sought, alongside aggressive symptom and comfort management. This case underscores a crucial reminder for emergency medicine physicians, echoing Hippocrates himself: “Cure sometimes, treat often, comfort always.” While emergent diagnostic and treatment capabilities will continue to advance over time, the resuscitate-first paradigm of emergency medicine may unintentionally lead patients onto care trajectories that are not appropriate. It is essential to be prepared to pause, engage in transparent and thoughtful discussions with patients and their families, and swiftly determine their preferences for aggressive medical and surgical interventions or intensive comfort-focused care options. The mastery of primary palliative care skills, including delivering bad news, initiating goals of care discussions, and identifying patients who would benefit from early specialized palliative care resources, is fundamental for ED physicians to possess and adeptly employ [20,21].

## Conclusions

In summary, we present a case of concomitant AIS, STEMI, and PE to add to the literature. It is essential for physicians, particularly emergency medicine physicians who encounter these cases on the frontlines of care, to consider a possible diagnosis of MOTEC. The authors encourage the use of this term to streamline the communication and management approach for patients experiencing critical thromboembolic events simultaneously affecting multiple organ systems. Early consideration and recognition will allow providers to deliver necessary and timely therapy as appropriate, and to coordinate resources for definitive testing and management. Additionally, healthcare providers should be familiar with their facility's capacity to integrate palliative medicine early in the ED course when aggressive curative measures do not align with the goals of care established by the patient or their family decision-makers. As this case report reflects only one patient presentation, limitations exist in generalizing a very specific patient population, such as those with MOTEC. Like many acute conditions, MOTEC is not a final diagnosis but rather a clinical syndrome resulting from various thrombotic or embolic pathophysiologies such as cancer, hereditary hypercoagulability syndromes, DIC, or atrial thrombi capable of affecting multiple distal organ systems. Additionally, there is considerable variability among medical centers in their capabilities to manage MOTEC, and formal clinical practice guidelines for this condition are currently lacking. Future studies should continue to define ED and interventional treatment algorithms that may be adopted into practice for these complex patients, to provide prompt recognition, stabilization, and definitive care guidelines.
